# Zinc and Other Metals Deficiencies and Risk of Type 1 Diabetes: An Ecological Study in the High Risk Sardinia Island

**DOI:** 10.1371/journal.pone.0141262

**Published:** 2015-11-11

**Authors:** Paolo Valera, Patrizia Zavattari, Alessandro Sanna, Salvatore Pretti, Alberto Marcello, Carla Mannu, Clara Targhetta, Graziella Bruno, Marco Songini

**Affiliations:** 1 Department of Civil-Environmental Engineering and Architecture, University of Cagliari, Cagliari, Italy; 2 Department of Biomedical Sciences, University of Cagliari, Cagliari, Italy; 3 Centre for the Study of Diabetes Complications and Metabolic Diseases, St. Michele Hospital, Cagliari, Italy; 4 Department of Medical Sciences, University of Turin, Turin, Italy; CINVESTAV-IPN, MEXICO

## Abstract

**Background:**

Type 1 diabetes incidence presents a decreasing gradient in Europe from the Nordic countries to the Mediterranean ones. Exception to this gradient is represented by Sardinia, the second largest Mediterranean island whose population shows the highest incidence in Europe, after Finland. The genetic features of this population have created a fertile ground for the epidemic of the disease, however, as well as being strikingly high, the incidence rate has suddenly presented a continuous increase from the ‘50s, not explainable by accumulation of new genetic variants. Several environmental factors have been taken into account, possibly interacting with the genetic/epigenetic scenario, but there are no strong evidences to date.

**Methods:**

The present study investigated the hypothesis that geochemical elements could create permissive environmental conditions for autoimmune diabetes. An ecological analysis was performed to test possible correlations between the values of eight elements in stream sediments and type 1 diabetes incidence rate in Sardinia.

**Results:**

Analyses revealed negative associations between elements, such as Co, Cr, Cu, Mn, Ni, Zn, and type 1 diabetes incidence.

**Conclusions:**

The results suggest a possible protective role of some elements against the onset of the disease.

## Introduction

Sardinia has the second highest type 1 diabetes (T1D) incidence rate (44.8/100 000 person-years, 0–14 years,1989–2009) [1], in the world, with an annual increase of 2.12%. The existence of this geographical clustering [2] is rooted primarily in the genetic peculiarities of the Sardinian population, where one of the haplotypes most predisposing to T1D (HLA-DR3-B18) is very frequent [3]. However, other factors may have contributed to make Sardinia such a fertile ground for T1D and specially have caused the dramatic increase in its incidence over time. This temporal trend cannot be explained by a genetic variability accumulation, which would have required much longer time for having an impact on the Sardinian population, and may instead be explained by the effect of environmental determinants. Although various factors have been hypothesized to be involved, including diet and infectious diseases, evidences are still very limited.

As regards the dietary factors, among others, the lack of vitamin D or its receptor despite the considerable sunlight in Sardinia, the use of cow's milk (casein, lactoglobulin), the presence of nitrates in tap water have been considered. No correlations were found between the incidence of T1D and the factors listed above [4,5].

Several infectious agents have been associated with the onset of diabetes in Sardinia: Enterovirus (COX, EV), Helicobacter pylori infection, Mycobacterium avium subspecies paratuberculosis (MAP), Malaria (Plasmodium), Helminths intestinal infections. The “Hygiene Hypothesis” claims that the human genes have adapted during evolution to a constant and a secular exposure to infectious agents. A very fast decrease of exposure can give immunological imbalance and increased susceptibility to the development of autoimmune diseases. In Sardinia, the almost complete disappearance of parasitic diseases, and eradication of such diseases, malaria, polio and tuberculosis, have coincided with an increase in autoimmune diseases such as multiple sclerosis, Crohn's disease, celiac disease, chronic and autoimmune thyroiditis, and T1D (Autoimmune clustering). There are several pieces of evidence indicating that MAP infection is linked to T1D in Sardinian patients [6]. A possible role of malaria infection (endemic in Sardinia since 2000 years B.C.) in determining the temporal increase of T1D incidence has been postulated, as just after its eradication in the 1950, immune-mediated disease began increasing precipitously. However, an overlap between areas with high prevalence of T1D and areas infested by malaria, has not been demonstrated thus far [5]. Helminths intestinal infections over the last four to five decades are disappearing in industrialized societies. Helminths and their eggs are probably the most potent selective stimulators of mucosal Th2 responses. Responses elicited by worms can modulate gut immune reactions to other parasites, bacterial, viral infections and several unrelated diseases. No conclusive evidence exists for this hypothesis [7]. Given these data there is no unifying hypothesis for the increase of T1D incidence in Sardinia, so it is mandatory to investigate other environmental factors.

Recently, studies have shown that blood abnormalities in metals might be associated with both T1D and T2D. Deficiencies of cromium (Cr) [8–12] has been suggested to increase lipid peroxidation, oxidative stress, and glucose levels in diabetic subjects [13,14]. Manganese (Mn) deficiency [8,9,15] would impair synthesis and secretion of insulin [16]. Cobalt (Co) also plays a relevant role as upregulator of Heme oxygenase-1 by cobalt protoporphyrin IX, thus playing a protective role in a context of autoimmunity, by preventing the expression of MHC class II molecules by antigen-presenting cells, so inhibiting activation and proliferation of autoreactive cells [17]. Recently, studies have focused the attention of researchers on the effect of copper (Cu) deficiency [18] and more extensively zinc (Zn) deficiency, as both these metals are involved in many cellular functions, including immune system homeostasis and insulin secretion. Interestingly, low zinc in drinking water was found to be associated with the risk of developing T1D during childhood in Sweden [19]. Therefore, it is relevant to assess whereas geographical variation in T1D risk within the Sardinia island are correlated with the geochemical distribution of such elements taking advantage of the existence in Sardinia of both a T1D incidence Registry in childhood and of an ongoing study on ore deposits prospecting, which started more than forty years ago, through the sampling of several environmental media supports and their geochemical analysis. This research activity has led to the creation of a large database, populated by more than 40k entries concerning soil, rocks and stream sediments. The stream sediments dataset used to conduct the present study, covers the whole Sardinian territory. Furthermore stream-sediment-type sampling, which is widely used for mineral prospecting, has the advantage of effectively representing the overall geochemical print of a wide portion of territory such as a stream basin.

In this study we performed an ecological analysis to assess whereas incidence of T1D within Sardinia was correlated with the geochemical distribution of elements, with a particular focus to Zn.

## Methods

### T1D Registry

As previously described [1], the registration of incident cases was performed according to the EURODIAB criteria. T1D was defined on the basis of clinical diagnosis of insulin-dependent diabetes by a doctor, excluding cases occurring secondary to other conditions. The date of onset was taken as the date of first insulin injection. The estimated completeness of ascertainment was 92% in period 1989–97, 90% in period 1998–2003 and 91% in period 2004–09, with no differences among genders and age-groups over the study period. Population data by sex and year of birth were provided by the Italian National Institute of Statistics (ISTAT, URL: http://www.demo.istat.it/). Age- and sex- specific incidence rates/100 000 person-years were calculated. The 1989–2009 T1D incidence rates for each municipality were calculated to assess the geographical risk distribution across Sardinia among children aged 0–14 years. Over the study period, 2371 incident cases were identified (1382 males, 988 females), giving an incidence rate of 44.8 cases per 100 000 person-years (95% confidence interval [CI] 43.1–46.7). Incidence rates varied within Sardinia from 40.6 cases per 100 000 person-years in the province of Sassari (North Sardinia) to 54.8 cases per 100 000 person-years in the province of Oristano (central-West Sardinia).

### Geochemical data

Sardinian Geochemical analytical data are mainly based on samples collected from previous surveys [20]. As reported by Marcello et al, considerable interest in geochemical prospecting has been shown in Sardinia, particularly over the last decades of the 20th century and many important prospecting campaigns have been officially funded, mainly by the Ente Minerario Sardo (EMSa) (Mining Institute of Sardinia), as part of its Programma Generale Straordinario di Ricerca (PGSR) (Extraordinary General Program of Research), and by the Ministero dell’Industria, Commercio e Artigianato (MICA) (Ministry of Industry, Trade and Small Industry) through a series of agreements, contracted with EMSa, for the improvement of basic research, but also by other organisations, such as universities, institutes and companies, operating in the mining sector and, particularly, in the field of environmental science. The University of Cagliari researchers, in this context, have collected over 2400 stream sediment samples, between 2000 and 2006, in order to complete the sampling of areas not considered yet by the previous sampling campaigns (Table 1).

**Table 1:** Samples collected by Geochemical Campaigns in Sardinia (adapted from 20).

1) Name of geochemical campaign	2) Organization	3) Executor	4) Year
Geochemical Strategic Prospecting of Sardinia	EMSa	BRGM	1972/74
Central-Eastern Area of Sardinia	MICA	EMSa-Progemisa	1987/90
South-Western Area of Sardinia	MICA	EMSa-Progemisa	1987/90
Granitoid Complexes of Sardinia	MICA	EMSa-Progemisa	1987/90
Palaeozoic Successions of North-Eastern Sardinia	MICA	EMSa-Progemisa	1987/90
Tertiary Volcanic Rocks of North-Western Sardinia—Area 1	MICA	EMSa-Progemisa	1991/93
Tertiary Volcanic Rocks of North-Western Sardinia—Area 2	MICA	EMSa-Progemisa	1991/94
Tertiary Volcanic Rocks of North-Western Sardinia—Area 3	MICA	EMSa-Progemisa	1991/94
Mineralisations in Hydrothermal Systems in Tertiary Volcanic Rocks of Sulcis	MICA	EMSa-Progemisa	1992/94
Indications of Gold and Base Metals in Tertiary Volcanic Rocks of North-Western Sardinia	MICA	EMSa-Progemisa	1994/97
Gold Mineralisations related to Shear Zones	MICA	EMSa-Progemisa	1994/97
Mineralisations in Palaeoplacers in Palaeozoic Formations of South-Eastern Sardinia	MICA	EMSa-Progemisa	1993/96
Various	Cagliari University	DIGITA	1998/2006
Various	Naples University	Dip. Geophysics and Volcanology	1999/2000

This study is based on stream sediment samples, which have a high variation of spatial density, with an average of approximately 2.2 samples/km^2^. Since 2006 the entire island territory was covered by analytical results by stream sediments samples using a regular grid of approximately 2 × 2.5 km, with at least one sample inside each rectangle (stratified-type sampling).

For this work a processing of the geochemical data was carried out, that allowed to establish the background of the main lithologies present, so that every abnormality (orebodies) was deleted during the processing itself. However, it is worth noting that the data are in any case affected by the genesis of the orebodies.

Most of the analytical data of the different sampling campaigns were collected, computerized, geo-referenced and verified by researchers at the University of Cagliari. Collected data and residual fractions of the samples are now available for environmental studies at the archives of the University of Cagliari.

All data are available in S1 Table.

### Geographical distribution of T1D incidence

T1D incidence rate and geochemical data were geo-referenced with a geographic information system (GIS). Homogeneous Areas (HA, n = 109) from lithological point of view were identified, where each HA includes one or more municipalities. T1D incidence rates were obtained by calculating the ratio between the number of incident cases over the twenty years period 1989–2009, and the number of residents (0–14 years) for each HA. Rates were normalized by the size of the area itself (Km^2^), given the geochemical rationale of this work [i.e., T1D incidence rate for a “i” HA, was calculated as HA_i_ normalized = (n cases_i_ / n residents_i_) * X_i_ Km^2^; for example, for HA number 1, HA1 normalized = (182 cases/450 127 residents)*710.24 Km^2^ = 0.287].

Demographic data were extracted from the Italian National Institute of Statistics—ISTAT web site (http://demo.istat.it/index.html), in which population data were obtained from Population Register Offices of each Italian municipality. Data available from the register are estimates in the intercensal period (ten years) and inferred by elaborating data concerning the last census (2011) and the demographic flows, such as births and deaths, in the decades considered.

All data are available in S1 Table.

### Elemental analysis procedures adopted in geochemical sampling campaigns

The samples collected in different sampling campaigns were analysed by using different analytical procedures, where the most commonly applied were AAS, INAA, ICP, XRF, and colorimetry for F. Although the heterogeneity of methods may generate unreliable sets of overall results, especially for the elements considered in almost all campaigns, i.e. Co, Cr, Cu, Mn, Ni, Pb and Zn, analysis duplication tests on a consistent number (1250) of stocked samples has demonstrated sufficient precision and accuracy [20].

*AAS (Atomic Absorption Spectroscopy)* it is a spectro analytical procedure for quantitative determination of chemical elements using absorption of optical radiation (light) emitted by free atoms in the gaseous state. This method was used until 1974 for trace elements, in particular, it was used in the first campaigns in Sardinia [21].

*INAA (Instrumental Neutron Activation Analysis)* was used between 1990–1994 for samples deriving from Tertiary volcanic lithologies. It was the standard analytical method until the introduction of ICP, for performing multi-element analyses with detection limits at the ppb level.

*ICP (Inductively Coupled Plasma)* was used for the sampling campaigns that started after 1990. This method was used to detect metals and other elements by ionizing the sample with inductively coupled plasma and then using a mass spectrometer to separate and quantify those ions.

In the present work, a total of eight elements (Co, Cr, Cu, Mn, Ni, Pb, U, Zn) were considered. The AAS method was used for the following elements: Pb, Zn, Cu, Ni, Co, Cr, Mn on samples coming from about 45% of the Sardinian territory, while for the other part of the territory the analytical methods used was ICP-MS for all eight elements.

Analytical data from stream sediments samples were chosen because they are the most representative, among the different sampling media, relatively to human health. Actually this kind of samples is ideal to determine geochemical conditions of broad zones and/or entire alluvial basins [22].

### Statistical analyses

The variables considered in this work are typical regionalized variables, as defined by Geostatistics, i.e. statistical variables that are function of the studied territory. These variables are the result of an unknown, but elevated, number of comparatively simple functions, in turn dependent from the spatial parameters of the territory, that most of the times could be described through Algebraic Analysis. However, only a comparatively small number of these functions can be satisfactorily defined, and in any case the preponderant number of the unknown and/or unsatisfactorily defined functions makes the resultant regionalized variable not definable analytically. As an example, if we consider the comparatively simple regionalized variable “size of quartz grains in the soil” in a given area, one of the comparatively simple functions that one immediately conceives as a component of this variable is “acclivity” (in the following indicated with a); in the elementary case of a flat surface, regularly dipping in the whole considered area, the analytical expression of this function is satisfactorily given by: a = constant. But immediately one also conceives a set of much less simple functions: abundance of quartz grains in the local rocks, resistance to mechanical erosion of these rocks, resistance to chemical leaching of the same rocks, formation of secondary quartz, thickness of the soils, local annual cycle of the rains, detrital transport from the surrounding areas, etc. Not all of these functions are really simple and easily described analytically, and the list of possibly influencing functions is still long.

Thus, Geostatistics concludes that the analytical expression of functional relations between two regionalized variables cannot be found, and the covariance only can show any relation among them. A quantitative assessment is given by the correlation coefficients, that may vary between -1 and 1, passing through 0. A value near 0 indicates the absence of relations, while a value more or less significantly approaching -1 or 1 indicates a more or less clear increase/decrease or increase/increase, respectively.

As a result of what above stated, correlation matrices and regression analysis were performed in this study in order to evaluate the correlation between T1D incidence distribution and geochemical data. Results are shown as R correlation index and level of statistical significance. A P value below 0.05 was considered as statistically significant. A correlation analysis was also conducted separately by gender.

Statistically significant positive correlations between geochemical data and T1D incidence rates were interpreted as potential predisposing factors to T1D, whereas statistically significant negative correlations were interpreted as potential protective factors.

### Validation of the correlation method

In nature, depending on the geological characteristics, some elements are highly correlated, and this behaviour has always been used in the mineral prospecting field in order to identify ore bodies. Indeed, sometimes the main elements (minerals of the ore body) are not easily detectable, due to their relative "geochemical immobility" or to the difficulties that may be encountered for their analytical determination (too high instrumental detection limits, for example). In these cases, it is useful to test other elements, geochemically more mobile, present in the same ore body, which are in association with the main elements. These elements are defined as "pathfinder" and have characteristics similar to the main elements but with a higher relative mobility during the geological processes that affected the ore body. Their association relationship can be considered constant throughout the geological context in which these elements are associated, so that a high content of a main element well matches with a high content of the indicator element (pathfinder) and vice versa. In general, some elements show the same association characteristics for a wide range of geological conditions, so that the knowledge of a territory under both geology and economic geology point of view allows to better evaluate the degree of association among the various elements.

Therefore, we examined the correlations between the analytical data of the considered elements to test the robustness of the correlation analysis.

## Results

### Pathfinder association results

As reported in Table 2, our analysis showed statistically significant correlation coefficients between the elements typically associated, for both ore bodies and lithologies, in clusters or spread, on the entire territory of Sardinia.

**Table 2:** Correlation coefficients between geochemical elements.

Associated elements	R	P value (two-tailed)
Pb—Zn	0.815	4.22x10^-27^
Cr—Co	0.811	1.22x10^-26^
Cu—Co	0.737	6.48x10^-20^
Zn—Cu	0.677	5.82x10^-16^
Cu—Mn	0.577	7.65x10^-11^
Zn—Co	0.534	2.28x10^-9^

### Correlation analysis between T1D incidence distribution and geochemical data

The correlation matrix between geochemical data and T1D incidence rates showed statistically significant negative correlations with Cu, Zn, Mn, Co, Cr and Pb.

Table 3 shows the correlation analysis between geochemical elements and T1D incidence rates. Apart from Ni and U, all other elements were significantly correlated with risk of T1D, particularly Co and Zn (two-tailed P value).

**Table 3:** Correlation coefficients between geochemical elements and T1D incidence rates,

Element	R	P value (two-tailed)	P value (one-tailed)
Co	-0.260	6.30x10^-3^	3.20x10^-3^
Cr	-0.242	1.11x10^-2^	5.61x10^-3^
Cu	-0.349	2.00x10^-4^	1.00x10^-4^
Mn	-0.306	1.35x10^-3^	6.75x10^-4^
Ni	-0.196	n.s.	n.s.
Pb	-0.230	1.16x10^-2^	8.00x10^-3^
U	-0.142	n.s.	n.s.
Zn	-0.332	4.20x10^-4^	2.10x10^-4^

n.s.: not statistically significant

Tables 4 and 5 show the results of the correlation analyses performed separately by gender (4 males, 5 females). No sex-specific effects were observed, although the correlations seem more pronounced in females.

**Table 4:** Correlation coefficients between geochemical elements and T1D incidence rates in males.

Element	Males		
	R	P value (two-tailed)	P value (one-tailed)
Co	-0.202	3.52x10^-2^	1.76x10^-2^
Cr	-0.201	3.61x10^-2^	1.80x10^-2^
Cu	-0.283	2.87x10^-3^	1.43x10^-3^
Mn	-0. 317	8.78x10^-4^	4.39x10^-4^
Pb	-0.166	n.s.	4.20x10^-2^
Zn	-0.258	6.76x10^-3^	3.38x10^-3^

n.s.: not statistically significant

**Table 5:** Correlation coefficients between geochemical elements and T1D incidence rates in females.

Element	Females		
	R	P value (two-tailed)	P value (one-tailed)
Co	-0.339	3.11x10^-4^	1.55x10^-4^
Cr	-0.288	2.39x10^-3^	1.19x10^-3^
Cu	-0.353	1.67x10^-4^	8.33x10^-5^
Mn	-0.266	5.62x10^-3^	2.81x10^-3^
Pb	-0.180	n.s.	3.05x10^-2^
Zn	-0.273	4.08x10^-3^	2.04x10^-3^

n.s.: not statistically significant

**Fig 1:**
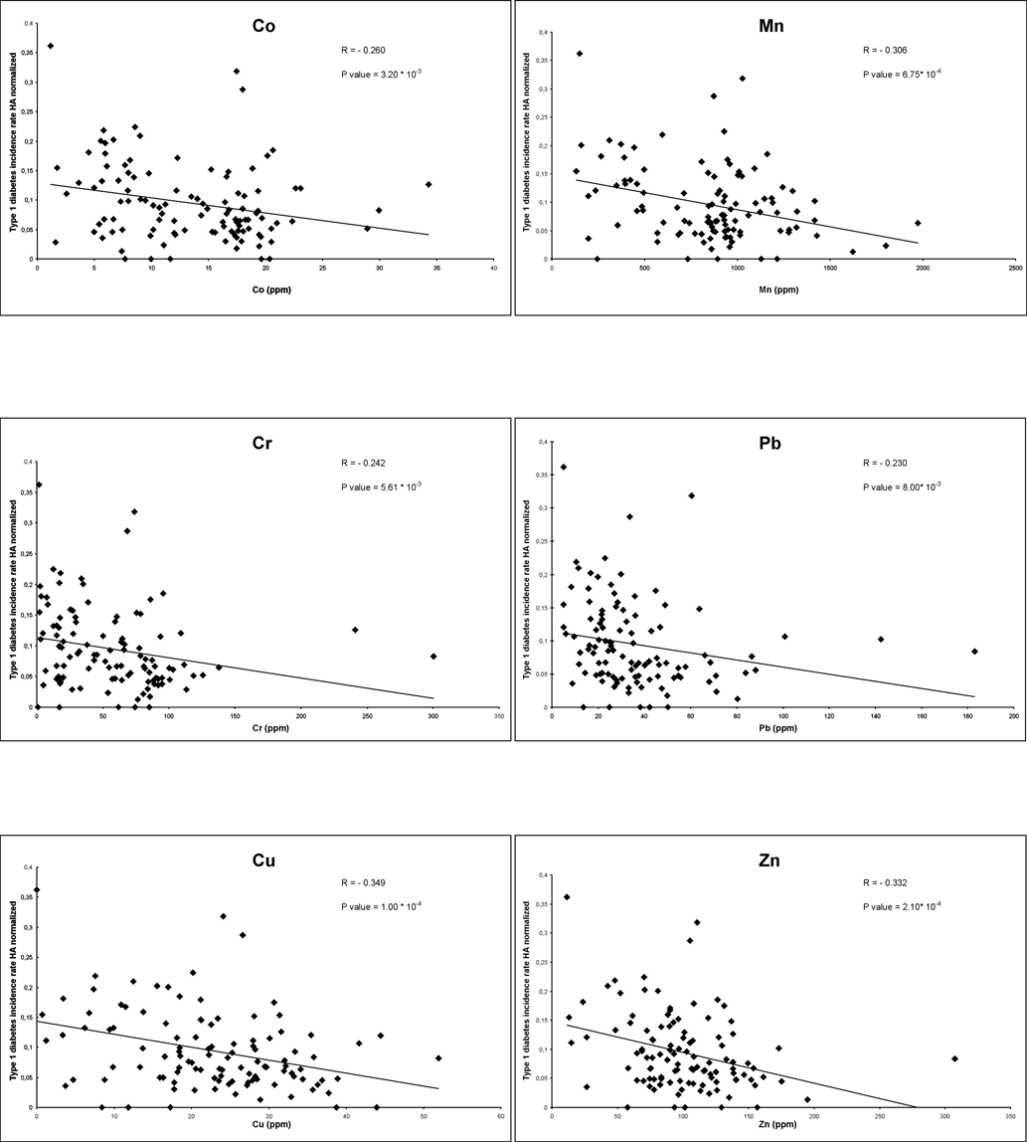
Regression analysis between T1D incidence rate and Co, Cr, Cu, Mn, Pb, Zn. On the X axis the geochemical elements value (ppm) is reported; on the Y axis is reported T1D incidence rate normalized by Homogeneous Areas (HA). For each element the correlation coefficient, R, and the respective P value are shown.

Each statistically significant correlation was analysed also through linear regression, as shown in Fig 1.

**Fig 2:**
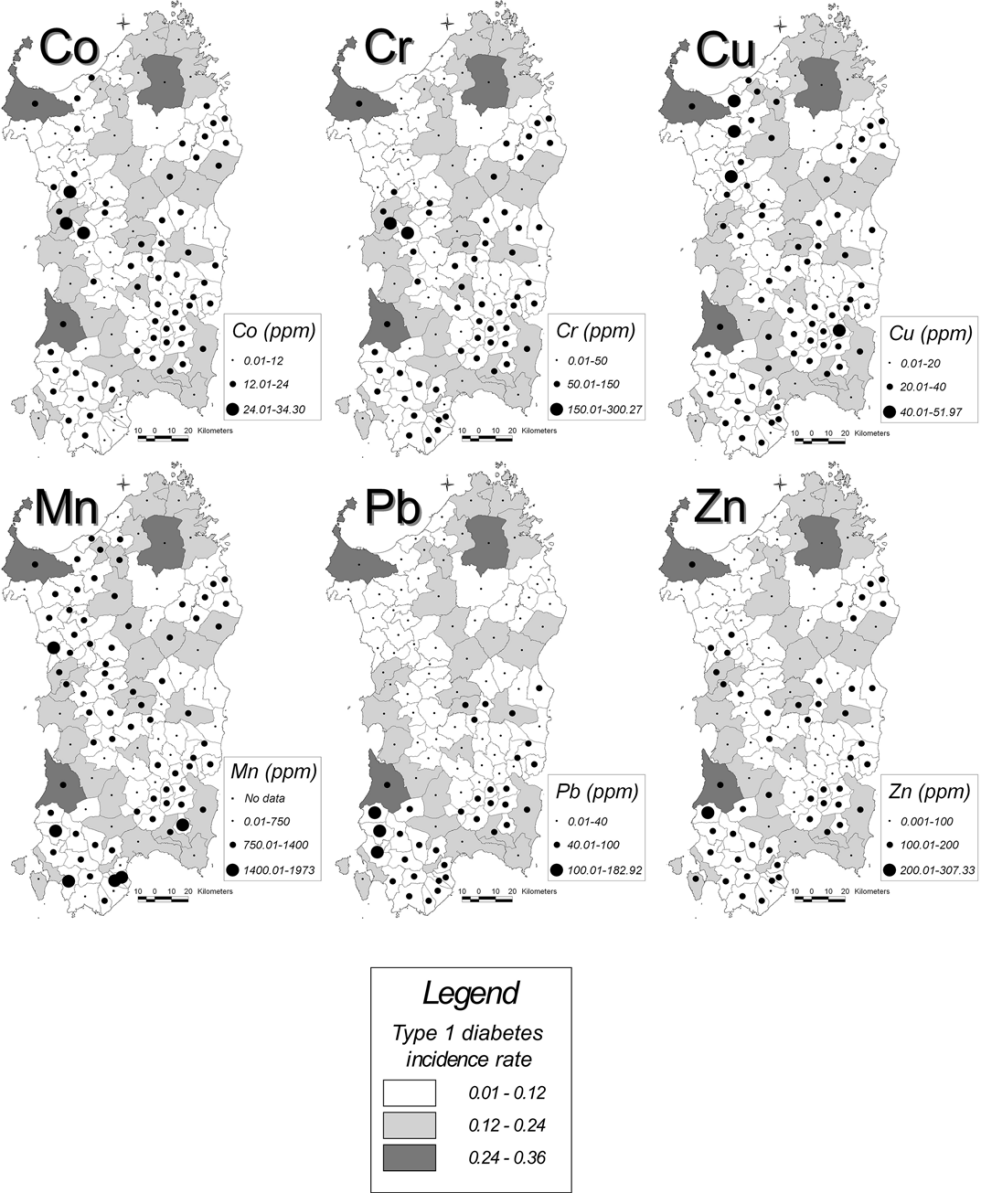
(A and B): Spatial distribution of T1D incidence rate and Co, Cr, Cu, Mn, Pb, Zn values in Sardinia. The results relate to 109 Homogeneous Areas (HA) identified from the lithological point of view. T1D incidence rate is represented by a chromatic scale, dividing values into three ranges, as indicated in the legend. Circles size symbolizes the amount of metal (ppm) detected in stream sediments samples, according to three ranges listed in the legend.

Fig 2 illustrates the spatial distribution of T1D incidence rates (chromatic scale) and Co, Cr, Cu, Mn, Pb, Zn values (circles).

## Discussion

The results of our ecological study showed that the geographical distribution of T1D incidence rates in Sardinia was correlated with elements distribution in stream sediments. Indeed, a statistically significant negative correlation was found between incidence rates and Co, Cr, Cu, Mn, Pb and Zn among 109 homogeneous areas. Soil contamination is a relevant problem in Sardinia. Besides having a very long history in mining [23], in recent decades Sardinia has begun to suffer the rising effects of the anthropic impact on health to such an extent that the soils in some areas, or entire districts, are particularly enriched of harmful elements.

In this study an ecological approach has been used to verify whether any considered element was differently distributed all over the island, where T1D has reached a pandemic pattern, although with heterogeneities within it (Fig 2), with the highest risk areas in southern and eastern parts of the island, and the lowest risk areas in the north-eastern part [24].

**Fig 3:**
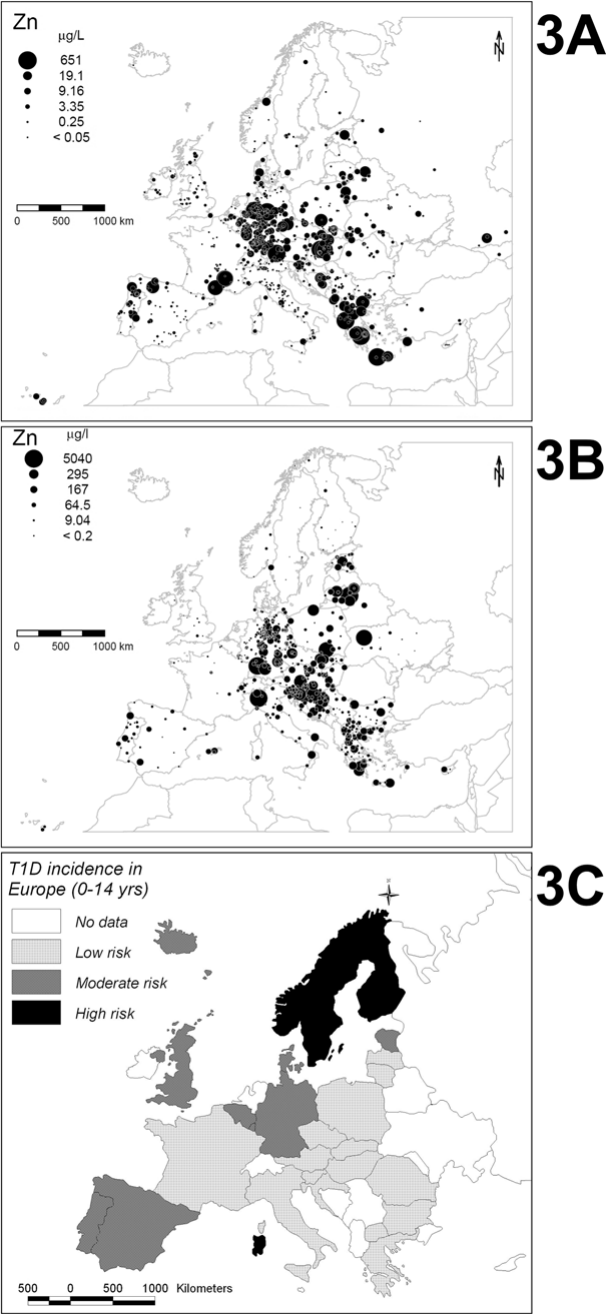
European spatial distribution of Zn and T1D incidence rate. **(A):** European spatial distribution of Zn content in bottled water (ug /L); circles size symbolizes the amount of Zn found, according to the legend reported. Reprinted from (25) under a CC BY license, with permission from Borntraeger Science Publishers, original copyright 2010. **(B):** European spatial distribution of Zn content in tap water (ug /L); circles size symbolizes the amount of Zn found, according to the legend reported. Reprinted from (26) under a CC BY license, with permission from Elsevier Publishers, original copyright 2015. **(C):** Distribution of T1D incidence rate (0–14 years) per 100.000 person-years, in several European countries, illustrated by means of a chromatic scale (see legend) that defines the high-risk countries (black), moderate-risk countries (dark gray), low-risk countries (light gray), unknown risk countries (white). This figure, generated for this manuscript for illustration purposes only, is based on T1D incidence rates reported in (27, 28).

Our findings are consistent with a Swedish study showing that low zinc in drinking water was associated with higher T1D risk in children [19]. Moreover, it appears very interesting to compare Zn values found in bottled (Fig 3A) [25] and tap waters (Fig 3B) [26], and T1D incidence (Fig 3C) [27,28] in Europe. It seems evident that water from countries with very high T1D incidence, as Scandinavia and UK, are particularly poor in Zn. Although our results are based on ecological analyses of data, they are consistent with current knowledge on the biological role of Zn for maintaining the proper functioning of many physiological processes. Zinc is a fundamental element in our cells, being an essential ligand for approximately 20% of the cell protein equipment. In particular, it binds to Zn transporters, metallothionines and zinc-finger proteins, mostly belonging to transcription factors. From a functional point of view, zinc is involved in the mechanisms underlying cellular movements, in the development of connective tissue, bone and teeth, in cell growth and endocrine regulation, cytokine production and immune responses [29]. Since the focus of this article, we can consider Zn-T8 as example of zinc carriers, transporter specifically expressed on the membrane of pancreatic beta cells, in which zinc is important for the formation of granules containing insulin, in particular for its crystallization in the secretory vesicles. The polymorphism rs13266634 (C > T), which causes R325W amino acid substitution, predisposes to diabetes impairing the proper insulin secretion and unbalancing the pro-insulin / insulin equilibrium in favour of pro-insulin [30]. It has been recently shown that zinc supplementation appears to differently affect the early insulin response to glucose, according to rs13266634 genotype, and could be beneficial for diabetes prevention and/or treatment for some individuals based on SLC30A8 variation [31]. With regard to the immune system, experiments in animal models have shown that zinc deficiency is associated with disorders of various nature that involve either a reduction in certain cytokines production and an altered cell-mediated response (macrophages, B and T cells, especially Th17 cells), increasing susceptibility to infections and autoimmunity [32–34]. This has been demonstrated both in the rheumatoid arthritis model, collagen-induced arthritis and in mice affected by experimental autoimmune encephalomyelitis -EAE- (animal model for multiple sclerosis) [33]. In particular, it was observed that a zinc deficiency induces thymic atrophy and lymphopenia, impairs cell responses and antibody-mediated and prolongs states of infection. A zinc deficiency also increases the eosinophilic inflammation and the risk of asthma. Furthermore, zinc chelating inhibits the release of histamine, cytokine production and secretion of lipid mediators (effects recovered by zinc integration) [35,36]. Therefore, zinc plays an important role in both innate and acquired response. It plays a significant role on the biological activity of thymulin (thymic hormone) and in the synthesis and release of various cytokines, such as IL-1 and IL-2 and a zinc deficiency leads to a depressed activity of thymulin, a reduced activity of natural killer cells, the CD4 + T cells, with decreased production of IgA, IgM and IgG [37].

Furthermore, zinc is an important neurotransmitter [38] and also a fundamental second messenger involved in the transduction of many cellular signals [39]. Finally, zinc is a micronutrient essential to the correct functioning of DNA methyltransferase (DNMT), therefore to the correct supply of methyl groups by which these enzymes regulate gene expression, by binding methyl groups to certain cytosines in the DNA filaments, particularly at the level of CpG islands located near gene promoters [40]. In animal models, it has been shown that zinc deficiency may be responsible for an intrauterine growth retardation, likely because of hypomethylation of certain loci, with consequent over-expression of key genes regulating beta cells development [41].

In terms of bioavailability, the major sources of zinc in the diet include: milk, red meat, liver, poultry, fish, oysters, crabs, cereals, legumes, tubers, nuts, vegetables. This bioavailability, however, is lower in cereals, legumes, tubers and vegetables, because of their content of phytate, lignin and fibers, which form insoluble complexes with zinc, compromising its intestinal absorption [42]. It was observed that zinc supplementation may decrease the risk of T2D but the effect is more evident by administering supplements, rather than with the diet [43]. By contrast, excessive levels of zinc can compromise the immune response, for example by increasing infection rates and durations, similar to those observed with zinc deficiency [44].

It is very interesting to note that different elements negatively associated with risk of T1D (such as Co, Cr, Cu, Mn, Zn) are typically present in Si-poor lithologies. In a recent study we suggested that these lithologies (i.e. mafic and ultra-mafic rocks) could be involved in reducing the risk of T1D [45]. However, it should be highlighted that the number of elements considered in the present study and in the previous one is quite limited and further studies are needed to better point out differences between Si-rich and Si-poor environments.

With regards to Pb, literature data are quite contrasting about its possible role on T1D risk. It is important to consider that natural associations between chemical elements may generate false positive results. Indeed, the high correlation between Pb and Zn, due to typical association on main ore deposits of Sardinia, may have played a role in making Pb as a confounder.

Copper deficiencies, and consequently deficiencies of proteins and enzymes binding this metal and requiring it to perform their function, are associated with a greater severity in the EAE model [18]. Indeed, certain metal-proteins, as the metallothioneins, play a neuroprotective role in the pathogenesis of EAE and a regulatory role in cell growth and in the maintenance of immune system homeostasis [46]. Cu, particularly as copper sulphate, also seems to exert beneficial effects in T1D, both directly by reducing free radicals amounts, and by lowering glucose levels in the blood [47].

We also found a statistically significant negative correlation between T1D incidence rates and Cr, Co and Mn. These results are consistent with previous data showing a deficiency of Cr and Mn in the blood of patients with T1D and T2D [8–11,15], and a protective role of Co towards autoimmunity [17].

In ecological studies the unit of observation is the population or community rather than the individual. Indeed, no data on blood concentration of examined elements were available in incident children with T1D in Sardinia included in this study. However, our results should be considered as hypothesis-generating rather than hypothesis testing study. If our results will be confirmed through proper observational studies comparing Zn concentration in blood of incident diabetic children, and in control children, they could support an intervention trial in high risk populations, such as the Sardinian one, to prevent and potentially even improve the natural course of the disease through Zn supplementation early in life.

## Key Messages

- This is the first population-study linking environmental geochemical elements exposure with risk of type 1 diabetes.
- This study suggests that some elements, typical of Si-poor lithologies, can play a protective role against the disease.
- If confirmed and appropriately tested by intervention trials in high risk populations, these results may suggest zinc and/or other metals as micronutrients supplementation in children at risk for type 1 diabetes.

## Supporting Information

S1 Table (XLS)
